# Journalists in a circular economy: Stakeholders’ engagement in the media discourse on single-use plastics during the COVID-19 pandemic

**DOI:** 10.1016/j.heliyon.2024.e36299

**Published:** 2024-08-17

**Authors:** Aleksandra Krawczyk, Alicja Goc, Airis Pellegrini, Natalia Jaguszewska, Brenda Olivos Salas, Michał Bukowski, Małgorzata Grodzińska-Jurczak

**Affiliations:** a Jagiellonian University, Doctoral School in the Social Sciences, Faculty of Geography and Geology, Institute of Geography and Spatial Management, Gronostajowa 7, 30-387, Kraków, Poland; b Jagiellonian University, Institute of Environmental Sciences, Gronostajowa 7, 30-387, Kraków, Poland; c Jagiellonian University, Institute of Linguistics and Translation Studies, al. Mickiewicza 3, 31-120, Kraków, Poland; d Jagiellonian University, Institute of Journalism, Media and Social Communication, ul. prof. Stanisława Łojasiewicza 4, 30-384, Kraków, Poland

**Keywords:** Media discourse, Environmental journalism, Single-use plastics, Circular economy, COVID-19

## Abstract

The world faces an alarming plastic waste problem. The volume of plastic waste is rapidly and continuously increasing, mainly due to the single-use plastics overconsumption, whereas its recycling and utilization leave much to be desired. Despite the negative effects of plastic on the environment and public health, the COVID-19 outbreak shifted the public attention away from the environmental issues, potentially giving space for extended lobbyism by interest groups and industry to delay or even prevent legislation to combat plastic pollution.

Our study aims to understand how the media discourse on single-use plastic (SUP) in particular, evolves in the course of the pandemic. How it vary across EU Member States? For this purpose, we specifically analyse plastic-related articles in major prestigious daily newspapers published between June 2019 and June 2021 in four EU Member States: Germany, France, Italy, and Poland, as countries with different levels of sustainable transition to form a representative model of an European context. Additionally, between November 2022 and January 2023, we conducted a series of interviews via Google Meet, with journalists who agreed to be asked on the plastic issues they upraised.

Our analysis initially covered 1076 articles, out of which 198 articles were rejected due to non-compliance with the subject or repetition, leaving 878 articles forming the database for eventual analysis. Specifically, we outline a key impact of the COVID-19 pandemic followed by a clear evolution on the number of plastic-related articles, on related stakeholder engagement, and the focus on specific SUP items. Moreover, we address a research gap - presenting a media portrait of different types of SUP in more details and highlighting the significance based on several culturally and linguistically very different countries within a single supranational state (EU).

A clear trend reversal towards an informed knowledge circulation across the circular economy model of single-use plastics is ultimately essential to develop sustainable solutions to reject the disposable culture, stop the waste of natural resources, and reduce the consumption of oil or gas for plastic production and thus protect the climate.

## 1 Introduction

Just a few decades ago, individually wrapped goods might have irritated us, whereas today we seem to have become accustomed to the convenience it provides. Requiring minimal effort from customers when purchasing goods or services has become crucial to market success [1,2]. Single-use plastic (SUP) items are durable, inert, and protective, widely used for packaging, hygiene products, and more [3,4]. Plastics offer cost-effective production and improved product performance, by reducing transportation costs and the carbon footprint associated with distribution. Owing to their high thermal and electrical insulation properties and long service life, plastics extend food shelf life, reducing waste. Plastic bottles efficiently deliver water to areas with scarcity [5]. Plastic has numerous advantages from this perspective.

The widespread use of plastics in everyday life obscures their significant negative impacts. According to recent studies, humanity produces 450 million tonnes of plastic waste annually, set to double by 2045 [6,7]. Macroplastic pollution eventually degrades into micro- and nanoparticles [8] resulting in a major threat to natural ecosystems [3,[9], [10], [11], [12]] and human health [8,13,14]. Plastic, with its low degradation rate (approximately a thousand years), is considered virtually irreversible pollution [[15], [16]].

The European Parliament addressed the plastic threat through the implementation of EU-wide rules on single-use plastic products (cotton buds, plastic cutlery, plates, straws, cups, stirrers, balloon sticks, expanded polystyrene [EPS] or ‘polystyrene’ food packaging and beverage containers). The primary goal of the Directive (EU) 2019/904 of the European Parliament and of the Council of June 5, 2019 on the reduction of the impact of certain plastic products on the environment is to encourage the adoption of sustainable and affordable alternatives, such as reusable glass and metal options. Under this directive, retailers must use these alternatives or use plastic products made from recycled materials. While existing disposable product stocks can be sold, new products must meet stricter labelling requirements and require producers to raise public awareness [17,18].

Before 2020, the world was concentrated on mitigating SUP usage through technical (such as waste recycling) and behavioural strategies [8,9]. The recent coronavirus pandemic has drastically altered perceptions of plastic products, particularly SUPs. The global consumption of disposable protective items markedly increased since the pandemic's onset, while global efforts to curb consumption have waned [8,19]. Due to the pandemic, the plastic threat became a behavioural problem; societies reverted to single-use items, notably in nations with high GDPs and heightened environmental awareness, as a means of health protection [20]. In consequence, the urgency of addressing sustainable and effective plastic waste strategies has been underscored [21,22].

Consequently, the problem is gaining increased societal attention [13]. Public opinion shapes human behaviour, yet human behaviour remains the primary source of plastic pollution. On the other hand, a substantial increase in public knowledge about environmental impacts can lead to behavioural changes [23]. Successful communication requires concise messages. In the context of education and knowledge transfer, shorter attention spans and the desire for instant gratification make it difficult for individuals to deal with complex content in a concentrated manner [24,25].

The quality press plays a crucial role in disseminating evidence-based knowledge as journalists obtain, verify, and contextualise information so that their audience can understand. However, environmental journalism faces challenges arising from what media scholars call “media logic” [26]. This concept refers to the production requirements of today's media and, in particular, to the appropriate message format. Three issues are especially challenging for environmental journalism: event-driven coverage, news cycles, and newsworthiness [27].

While traditional media prioritize distinctive events, key environmental issues often lack the immediate dramatic impact necessary for such framing. The Exxon Valdez oil spill and Fukushima disaster are notable events, while plastic pollution is not. Environmental issues often do not align with the 24-h news cycle and lack news value [28], such as proximity, timeliness, negativity, and unexpectedness. Therefore, they may not receive adequate coverage.

Thus, the subtle nature of environmental processes does not meet the requirements of contemporary news formats. The concept of the ‘environmental beat’ helps to reconcile the logic of the media with scientifically framed environmental problems [29]. It serves as an interpretative framework crafted by journalists, incorporating a specific timescale, that enables the presentation of complex processes as definable events with appropriate newsworthiness, suitable for a journalistic story.

One example is the problem of the Kiribati islands, which are at risk of being completely submerged by rising sea levels in the coming decades. Global warming is thus framed as a narrative of personal tragedy for the people of Kiribati, allowing journalists to move from an abstract process to concrete events in a way that is engaging and persuasive to readers yet scientifically accurate and evidence-based.

Researchers agree that how an issue is represented in the symbolic environment [30], which includes media discourses, has an impact on the chances of solving the problem. Despite this, there remain few empirical studies documenting how the problem of single-use plastics is actually portrayed in the media. Existing studies focus on plastics in general [31,32], plastic pollution [33], and microplastics [23,34]. Our study focuses in detail on SUP and on different products of SUP. Moreover, most published studies focus on either a single country [35] or a group of culturally similar countries [36]. Against this background, our case presents culturally and linguistically very different countries within a single supranational state (EU). Our study also aims to examine the impact of the COVID-19 pandemic on the dissemination of information about SUP, which is also considered in [21,32,37].

**Fig. 1:**
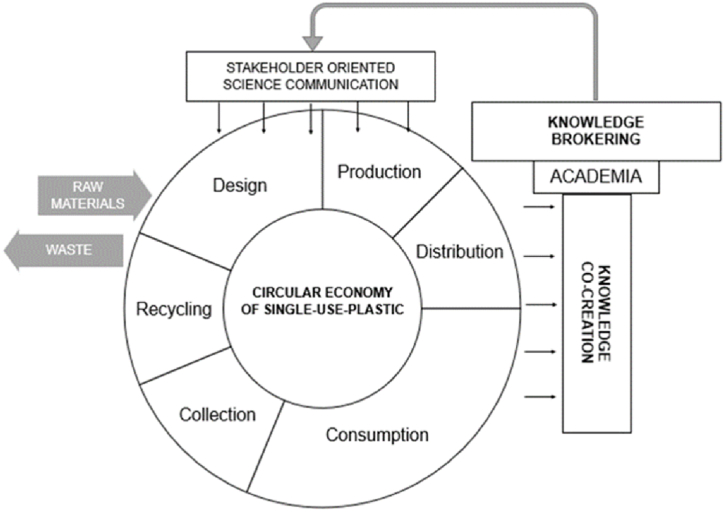
Science communication as collaborative knowledge co-creation and brokering within a circular economy of single-use plastic. Author's elaboration based on the concepts of Circular Economy in Ref. [38] and Science Communication and knowledge brokering in Ref. [39].

Here, we employ a polycontextualized model of the SUP Circular Economy (CE) (Fig. 1.) to understand the interlinkage between European journalists (media) and other CE stakeholders (i.e., product designers, producers, end consumers, waste management representatives, recyclers) to co-create knowledge for effective science communication.

With the proposed study, we aim to understand how European journalists may act as knowledge brokers to the knowledge circulation within a CE of SUP and eventually contribute to individual transformations among the public. To achieve this goal, we ask the following research questions: (1) How does the SUP related media discourse evolve in view of the COVID-19 pandemic? (1a) Which SUP items are of interest? (1b) Which stakeholders are being engaged? (2) What are current practices in environmental journalism? (2a) How independent are they in the agenda setting? (2b) Which stakeholder do they engage and how? (2c) Are there any conflicts of interest? (2d) How did the COVID-19 pandemic affected their work? (2e) What needs to be improved from the perspective of environmental scientists?

**Fig. 2:**
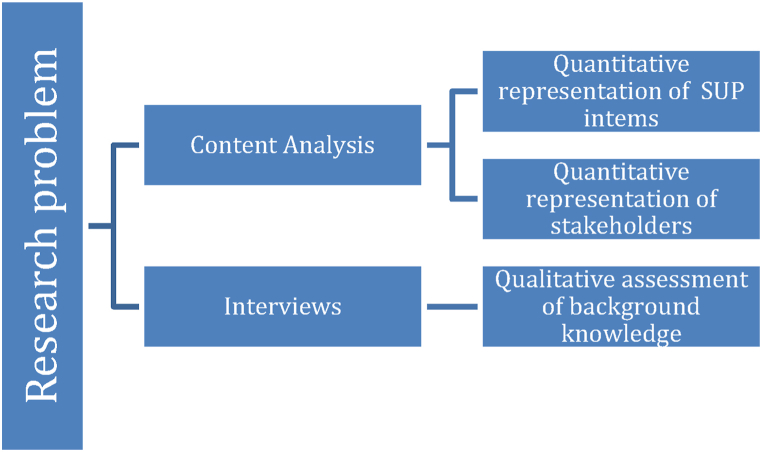
Diagram showing the methodological plan of the research.

The diagram of our methodological approach is shown in Fig. 2 below.

## 2 Materials and methods

### 2.1 Media discourse

The selection of research material aimed to enable a comparison of the mainstream public discourse about single-use plastics in four European countries: Germany, Poland, France and Italy. This choice was based on the TPI (European Commission, Transitions performance index 2021), as these countries showed different levels of sustainable transition to constitute a representative model for a European setting.

In each country, two prestigious general-interest dailies were selected for analysis. For Germany these were: Frankfurter Allgemeine Zeitung and Süddeutsche Zeitung, for Poland: Gazeta Wyborcza and Rzeczpospolita, for France: Le Monde and Le Figaro, for Italy: Corriere della Sera and la Repubblica. Comparing prestigious newspapers has a long-standing tradition in media studies [[40], [41], [42], [43]] and is one of the analytical strategies recommended in methodological textbooks [44]. The choice is based on the assumption that the prestige press (the legacy media) is crucial for ‘national conversations’ as it evokes, publicizes, and sustains in the media circulation particularly relevant political and social issues, setting the agenda among the public. As Langer et al. [45] recently demonstrated, this is also the case in times of hybridized media systems and despite the ubiquity of new media.

The digital archives of selected newspapers were searched. We identified all relevant articles published between June 2019 and June 2021 using specified keywords and phrases (see: Appendix A1). From the initial 1076 articles identified, 198 were excluded due to non-relevance or repetition, leaving 878 articles for analysis. We reviewed the selected articles focusing on two main categories: the SUP item of interest and the involved SUP stakeholders.

Publication quantity indicators were compared to the level of COVID-19 related restrictions in the authors’ countries. To this purpose, the Oxford Coronavirus Government Response Tracker project [46] served as a database of pandemic policies containing data from all over the world from January 21, 2020, constantly updated. Specifically, we used the Stringency Index, a measure that consists of nine response indicators, including among others: school and workplace closures, social distancing, travel restrictions and vaccination availability, that could have a significant impact on the work and private life of journalists. Each indicator is weighted according to its restriction level (strong or weak) and its geographic scope within the concerned country. The final stringency index score for a country on a given day is the average of all nine indexes, ranging from 0 to 100, with 100 being the most severe response [46].

**Fig. 3:**
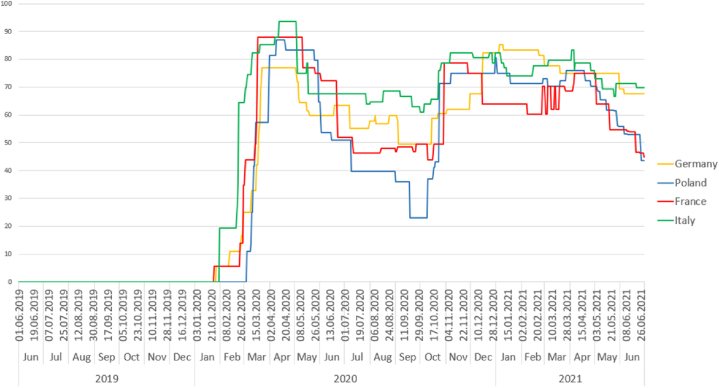
Oxford Coronavirus Government Response Tracker: Stringency Index for selected EU countries.

Fig. 3 illustrates the COVID-19 stringency index peaking exponentially across all countries around January/February 2020. It gradually declines around May/June 2020, notably for Poland, which sees its lowest drop around September/October 2020. The index rises around October/November 2020, remaining mostly stable until a general decline in April 2021, particularly noticeable in France and Poland. For this study, we used the average monthly scores of the stringency index.

We conducted statistical analysis using RStudio (2021.09.0 + 351 “Ghost Orchid” Release). In order to determine the shape of the distribution of the data, its skewness and kurtosis were tested. Skewness measures the asymmetry of a distribution, while kurtosis indicates if a distribution is heavy-tailed or light-tailed compared to a normal distribution [47]. The monthly publication data do not follow a normal distribution. Data on SUP items and stakeholder engagement in the articles are sparsely distributed and do not follow a normal distribution. Due to these characteristics, only non-parametric tests could be run.

To test correlation, Spearman correlation test was used, indicating the strength and direction of correlation between two variables [48,49]. Unlike its parametric equivalent, Pearson's correlation test, The Spearman test detects not a linear but a monotonic relationship between variables. A monotonic relationship, being less restrictive than a linear one, is identified when the value of one variable increases (positive correlation) or decreases (negative correlation) with the increase of the value of the other variable [48,50]. The correlation coefficient ρ ranging from −1 to 1, reveals the direction of the relationship. Another important difference between Pearson and Spearman correlation tests is that the Spearman test computes the correlation coefficient using the ranks of variables' values, not the actual values [50]. To further assess if the stringency index significantly impacts the other variable, we employed the Kruskal–Wallis test [49,51]. To account for the apparent positive correlation between the number of articles and the counts of mentioned SUP items/stakeholders, the counts of SUP items/stakeholders were divided by the number of articles.

### 2.2 Interviews

Interviews conducted as a part of this study took place via Google Meet between November 2022 and January 2023. Invitations to join interviews were sent via email or social media (Twitter, Linkedin, Facebook) to 113 identified authors who published at least two of the SUP related articles in our database. Ultimately, seven journalists agreed to participate in the interviews (3 from Poland, 2 from France, 2 from Italy).

Given their versatility and flexibility, semi-structured interviews are popular in social science research [52]. This method includes questions carefully selected from the literature review [53] serving more as a framework than strict instructions. Hence, the order of the questions was adjusted based on the interview flow and reactions of the respondents, i.e., in case of a desire to deepen a topic or to explore something perceived as important, the interviewers allowed the respondent to continue his/her thoughts. Following a brief introduction covering the project's aim, background, and current research, we asked respondents to describe their journalistic practices (*“Tell us about the process of article creation.”*) and stakeholders' engagement (*“How important is the engagement of different groups of stakeholders within a potential circular economy of SUP?”*, *“How do you perceive its responsibility in fighting the environmental crisis?”)*. One of the questions was on the topic of COVID-19 pandemic and its impact on journalism (*“How do you perceive the impact of COVID-19?“).* We also asked about the journalists' opinion on any problems or risks in their profession (*“Should anything be changed in journalistic practice to communicate knowledge?”)* and about their role in the opinion making process (*“What is the role of the public press in the opinion making process in society?”*). All questions were asked in English; however, respondents were allowed to answer in their native language to accommodate language barriers. With respondent consent, we recorded and, when necessary, transcribed and translated all conversations. Each interview lasted about 1 h and was conducted by at least two members of the research team. The total transcript time amounted to 7 h and 16 min.

## 3 Results

We present all gathered and carefully analyzed data below in the light of the research questions we posed. The organization of chapters and subchapters corresponds to the research questions (see the Introduction).

### 3.1 Articles review

**Fig. 4:**
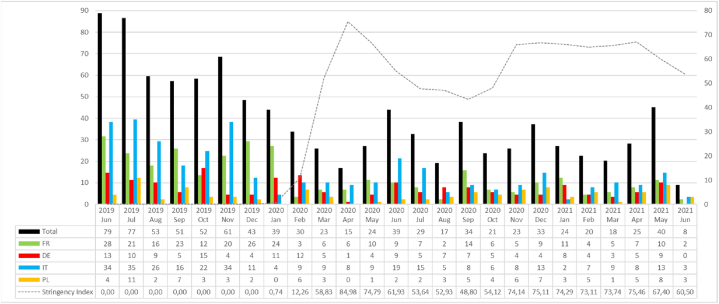
Number of SUP related articles vs. average stringency index for the selected countries.

We distributed the 878 investigated plastic-related press articles as follows: Germany 163: Frankfurter Allgemeine Zeitung (111), Süddeutsche Zeitung (52), Poland 96: Gazeta Wyborcza (45), Rzeczpospolita (51), France 286: Le Monde (87), Le Figaro (199), Italy 333: Corriere della Sera (173), la Repubblica (160). Fig. 4 presents the detailed distribution from June 2019 to February 2021 against the COVID-19 stringency index. The mean number of monthly published articles is 8.78. The highest mean number of monthly articles was in Italy (13.3), followed by France (11.4), Germany (6.52) and Poland (2.62).

We observed a significant negative correlation between the COVID-19 stringency index and the number of articles (∼p = 0.0095, ∼ρ = −0.26) (Appendix C.1). The Kruskal-Wallis test showed a significant impact of the stringency index on the number of articles (∼p = 0.034). At the country level, we found significant correlations for Germany (∼p = 0.0117, ∼ρ = −0.496), France (∼p = 0.0027, ∼ρ = −0.57) and Italy (∼p = 0.0051, ∼ρ = 0.54) (Appendix C.2). This finding was anticipated given the unique circumstances of the pandemic, which necessitated prioritizing pandemic-related issues. However, while statistically significant, the correlation is not strong enough to suggest that the media have almost completely abandoned the SUP issue.

#### 3.1.1 SUP items specification

Of the 878 articles, 293 (33.4 %) focused on plastic in general, 324 (36,9 %) on SUP in general, and 261 (29,7 %) on a specific sort of SUP. Considering that some articles mentioned more than one SUP item, we identified in total 314 cases of SUP item specifications for further analysis. The most frequently specified SUP items were bottles (115–36,6 %), followed by packaging (64–20,4 %), cups (23–7,3 %), masks (23–7,3 %), grocery bags (19–6,0 %), food containers (19–6,0 %), followed by straws (13–4,1 %), cutlery (10–3,2 %), trash bags (7–2,3 %), gloves (7–2,3 %). The least mentioned SUP items included fruits/veggies bags (5–1,6 %), laboratory utensils (4–1,3 %), plates (4–1,3 %), menstrual utensils (1–0,3 %). Details are reflected in Table 1.

**Table 1:** Number of articles related to plastic in general, SUP in general and specific SUP items, distribution of SUP specifications.

	Total	France	Germany	Italy	Poland
Number of articles	878	286	163	333	96
• Plastic in general	293	83	36	152	22
• SUP in general	324	63	85	129	47
• SUP item(s)	261	140	42	52	27
Sum of SUP specifications	314 (100 %)	170 (100 %)	46 (100 %)	67 (100 %)	31 (100 %)
Bottles	115 (36,6 %)	73 (42,9 %)	9 (19,6 %)	17 (25,3 %)	16 (51,6 %)
Packaging	64 (20,4 %)	41 (24,1 %)	5 (10,9 %)	18 (26,8 %)	–
Cups	23 (7,3 %)	7 (4,1 %)	6 (13,1 %)	6 (9,0 %)	4 (13,0 %)
Masks	23 (7,3 %)	16 (9,4 %)	–	6 (9,0 %)	–
Grocery bags	19 (6,0 %)	7 (4,1 %)	7 (15,2 %)	2 (3,0 %)	3 (9,7 %)
Food containers	19 (6,0 %)	10 (5,9 %)	3 (6,5 %)	1 (1,5 %)	5 (16,1 %)
Straws	13 (4,1 %)	6 (3,5 %)	3 (6,5 %)	4 (6,0 %)	–
Cutlery	10 (3,2 %)	3 (1,8 %)	3 (6,5 %)	4 (6,0 %)	–
Trash bags	7 (2,3 %)	2 (1,2 %)	2 (4,3 %)	3 (4,4 %)	1 (3,2 %)
Gloves	7 (2,3 %)	3 (1,8 %)	–	4 (6,0 %)	–
Fruits/veggies bags	5 (1,6 %)	1 (0,6 %)	3 (6,5 %)	–	1 (3,2 %)
Laboratory utensils	4 (1,3 %)	–	4 (8,7 %)	–	–
Plates	4 (1,3 %)	1 (0,6 %)	–	2 (3,0 %)	1 (3,2 %)
Menstrual utensils	1 (0,3 %)	–	1 (2,2 %)	–	–
Sum	314 (100 %)	170 (100 %)	46 (100 %)	67 (100 %)	31 (100 %)

In France, of the 286 articles, 83 (29 %) addressed plastic in general, 63 (22 %) SUP in general, and 140 (49 %) specific SUP items. Given that some articles mentioned multiple items, we identified 170 cases of SUP specification for further analysis. Bottles and packaging were the most common by a significant margin.

In Germany, of the 163 articles, 36 (22 %) focused on plastic in general, 85 (52 %) on SUP in general, and 42 (26 %) on specific SUP items. With some articles mentioning more than one specific SUP item, we identified 46 cases of SUP specification for further analysis. Bottles were the most frequently mentioned, followed by grocery bags and cups.

In Italy, of the 333 articles, 152 (46 %) addressed plastic in general, 129 (39 %) SUP in general, and 52 (15 %) specific SUP items. Given that some articles focused on more than one SUP item, 67 cases were identified for further analysis. Packaging and bottles were the most common.

In Poland, of the 96 articles, 22 (23 %) focused on plastic in general, 47 (49 %) on SUP in general, and 27 (28 %) on specific SUP items. With some articles showcasing more than one SUP item, 31 cases were identified for further analysis. Bottles were the most frequently mentioned SUP items.

**Fig. 5:**
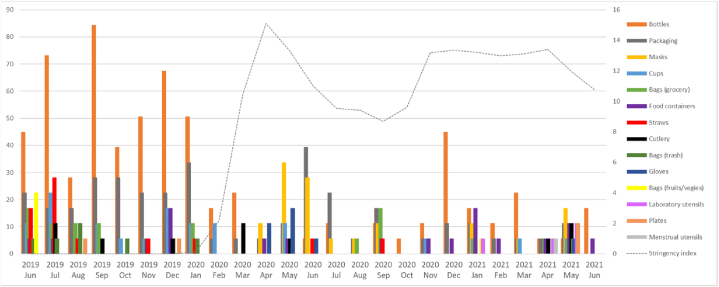
SUP item specifications in total vs. COVID-19 stringency index.

Fig. 5 shows illustrates the detailed distribution from June 2019 to June 2021 against the COVID-19 stringency index. Detailed data are in Appendix A1, with the graphical presentation of the countries (France, Germany, Italy, and Poland) in Appendix C1.

We found no significant correlation between the stringency index and the monthly count of SUP items mentioned per article (∼p = 0.64), though the average count dropped from 0.6 to 0.4 before and after the pandemic's outbreak.

For different types of single-use plastic items, we observed a significant positive correlation between the stringency index and the monthly counts of per article mentioned: masks (∼p = 0.019, ∼ρ = 0.46; the mean rose from 0 to 0.065 between the periods before and after the coronavirus pandemic outbreak); and gloves (∼p = 0.026, ∼ρ = 0.45; the mean rose from 0 to 0.027 between the periods) (Appendix C.3). We identified a significant negative correlation between the stringency index and the monthly counts of per article mentioned: grocery bags (p = 0.02746, ∼ρ = −0.44; the mean fell from 0.032 to 0.017 between the periods before and after the coronavirus pandemic outbreak); trash bags (p = 0.02072, ∼ρ = −0.46; the mean fell from 0.029 to 0.0036); bottles (∼p = 0.002, ∼ρ = −0.58; the mean fell from 0.22 to 0.082); and straws (∼p = 0.007, ∼ρ = −0.53; the mean fell from 0.042 to 0.003) (Appendix C.4). This suggests the pandemic and lockdowns eliminated or significantly reduced some everyday activities (such as normal shopping) but triggered others, such as the compulsory wearing of masks and gloves, leading journalists to focus on SUP items relevant to life under pandemic restrictions.

In France, we found a significant negative correlation between the stringency index and the monthly mentions per article: bottles (∼p = 0.0015, ∼ρ = 0.6); and straws (∼p = 0.022, ∼ρ = 0.45). In Germany, we identified a significant positive correlation between the stringency index and the monthly counts of laboratory utensils mentioned per article (∼p = 0.036, ∼ρ = 0.42), additionally, a significant negative correlation between the stringency index and the monthly mentions per article: grocery bags (∼p = 0.0003, ∼ρ = 0.66); and trash bags (∼p = 0.067, ∼ρ = 0.37. In Poland and Italy, we found no significant correlations between the monthly mentions of any SUP item per article and the stringency index.

#### 3.1.2 Stakeholders engagement

Additionally, 71.4 % (627) of the 878 articles cited or paraphrased at least one stakeholder group, yielding a total of 884 stakeholder engagements for further analysis: 248 (28,1 %) politicians, 234 (26,5 %) industry representatives, 130 (14,7 %) NGOs, 110 (12,4 %) scientists, 60 (6,8 %) plastic producers, 39 (4,4 %) recyclers, 31 (3,5 %) waste management representatives, 25 (2,8 %) end consumers, and 7 (0,8 %) designers. Table 2 presents these details, and Appendix B outlines the detailed distribution across the investigated period.

**Table 2:** Distribution of stakeholder engagement.

	Total	France	Germany	Italy	Poland
Articles	878	333	183	333	96
Cases of stakeholder engagement	627 (100 %)	393 (100 %)	212 (100 %)	157 (100 %)	122 (100 %)
Politicians	248 (28,1 %)	135 (34,3 %)	49 (23,1 %)	27 (17,2 %)	37 (30,3 %)
Industry	234 (26,5 %)	96 (24,4 %)	62 (29,2 %)	43 (27,4 %)	33 (27,1 %)
NGOs	130 (14,7 %)	60 (15,3 %)	20 (9,4 %)	39 (24,8 %)	11 (9,0 %)
Scientists	110 (12,4 %)	53 (13,5 %)	29 (13,7 %)	9 (5,7 %)	19 (15,6 %)
Plastic producers	60 (6,8 %)	12 (3,1 %)	29 (13,7 %)	14 (8,9 %)	5 (4,1 %)
Recyclers	39 (4,4 %)	15 (3,8 %)	4 (1,9 %)	10 (6,4 %)	10 (8,2 %)
Waste management	31 (3,5 %)	9 (2,3 %)	12 (5,7 %)	5 (3,2 %)	5 (4,1 %)
End consumers	25 (2,8 %)	13 (3,3 %)	4 (1,9 %)	7 (4,5 %)	1 (0,8 %)
Designers	7 (0,8 %)	0 (0,0 %)	3 (1,4 %)	3 (1,9 %)	1 (0,8 %)

Stakeholders were cited or mentioned 393 times in 333 French articles. Politicians and industry representatives were the most frequently cited stakeholder groups. Stakeholders were cited or mentioned 212 times in 183 German articles. Industry representatives and politicians were the most common. In 333 Italian articles, stakeholders received 157 citations or mentions. The most common were industry representatives, NGOs, and politicians. Stakeholders were cited or mentioned 122 times in 96 Polish articles. Politicians and industry representatives were the stakeholders most frequently mentioned. The graphic distribution for the different countries over the investigated period is presented in Appendix C2.

**Fig. 6:**
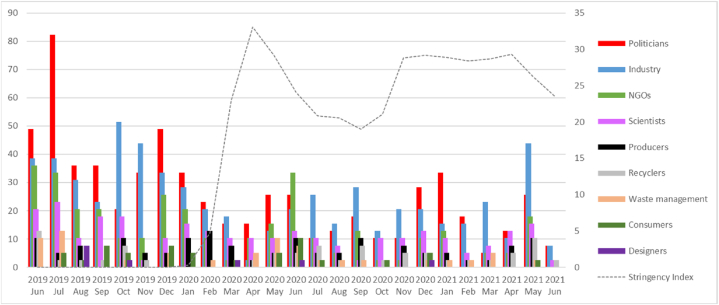
SUP stakeholders' engagement in total vs. COVID-19 stringency index.

Fig. 6 presents the identified cases of stakeholder engagement over the investigated period against the COVID-19 stringency index. We found a significant positive correlation between the stringency index and the monthly total count of stakeholder mentions per article (∼p = 0.038, ∼ρ = 0.42). The average mentions per article rose from 0.99 to 1.12 before and after the pandemic outbreak. A visual representation of this correlation is depicted in Appendix C.5. This increase was also observed, albeit insignificantly, in each country separately (Appendix C.6). The pandemic sparked a general increase in interest in stakeholders' and experts' viewpoints. Journalists sought comments and explanations for a complex and uncertain situation to relay to readers. The diversity of explanations led to a diversity of experts. Conversely, stakeholders in the media aimed to influence public opinion on the taken restrictive measures, which in the case of industry representatives or politicians was directly related to their interests as social actors.

A significant positive correlation exists between the stringency index and the monthly mentions per article between the stringency index and the monthly counts of per article mentioned: scientists (∼p = 0.0004, ∼ρ = 0.65; the mean rose from 0.098 to 0.16 between the periods before and after the coronavirus pandemic outbreak); and recyclers (p = 0.0112, ∼ρ = 0.498; the mean rose from 0.028 to 0.07) (Appendix C.7).

In Germany, a significant positive correlation was found between the stringency index and the monthly mentions of NGOs per article (∼p = 0.026, ∼ρ = −0.44). In France, Italy, and Poland, we found no significant correlations between the stringency index and any group of stakeholders mentioned monthly per article.

### 3.2 Practices in environmental journalism

#### 3.2.1 Profile of respondents, agenda setting, research process

Of the 113 journalists identified (each authoring at least two SUP-related articles in our database), seven journalists consented to interviews (three from Poland, two from France, two from Italy). For two interviewees, environmental topics are not a routine part of their job, for three, they serve as a secondary subject, environmental topics.

All respondents affirmed their involvement in the decision-making process concerning their topic selection. However, the editorial team and head editor also play a significant role in the process, often commissioning articles on specific topics. Journalists draw inspiration from various sources, including those closely related to their professional environment, such as press releases (“*there are press agencies that send us press releases*”; “*already prepared data which the journalist can then take and publish by simply changing a few things*”), studies (*“we have access to papers that are going to be published*”), reports (“*could be reports made by the various observatories*”). Additionally, interactions with individuals serve as a source of inspiration (“*discussions on the day-to-day life too*”; “*it can be created during speaking with friends*”; “*usually, I'm talking with somebody because of something*”; “*somebody calling you and saying, Oh, look, there is a guy who wrote an interesting book*”). For research, journalists rely on the Internet and reports as key information sources. They frequently initiate calls and, when necessary, arrange interviews with relevant stakeholders.

#### 3.2.2Stakeholders’ engagement

The decision to engage specific stakeholders primarily depends on the article's subject (“*it depends on the subject*”; “*it depends on the service I'm working on*”). There's a general inclination to present varied perspectives and opinions on an issue. To achieve this, they contact field experts, like scientists (“*mostly I contact scientists*”), NGOs or other organizations (“*NGOs are the most interesting source because their goal is not to sell products*”; “*NGOs have a lot of information, because they are digging in topics all the time*”; “*but also the president of an association*”), industry representatives (*“sometimes spokespersons from companies*”; “*I go directly to the plants that collect and process plastic materials*”), trustworthy politicians (*“politicians in the sense that they know something of what they are saying*”; “*they also are decision makers, so have a lot of information*”), and waste management organizations, particularly for environmental subjects (*“you can call your informants like some organization connected with waste management*”), citizens (*“go and interview people on the street”; “and normal people, you can also ask*”), or expert journalists in a particular field (“*also journalists from other countries*”).

Cooperation with stakeholders varies not only by type but also within each category. Respondents mainly described four types, namely.

(1) Companies – that are seen as available but often only when the article benefits them (“*sometimes would not answer or would give very general answers*”). Their approach also varies from one company to another (“*some companies are open, others are more difficult*”). Generally, they are not entirely transparent (“*directors and CEOs or companies which have spokespersons are the most problematic here*”).
(2) Scientists – that are generally considered accessible (“*researchers are very open, cooperative, helpful*”), and often well-connected to relevant issues (“*the scientists I call most of the time are very connected to the issues*”), though communication issues sometimes arise (“*some are too theoretical*”; “*they are making it very difficult to read, and they cover it with some words which make them feel safe*”).
(3) Politicians, especially ministries – that are often challenging to reach (“*it is extremely complicated to get feedback from the Directorate General of Health or the French administrations*”). However, some respondents find individual politicians readily available (“*politicians are quite open too*”), yet others report difficulties in contacting them (“*politicians are difficult to contact*”).
(4) NGOs – that are generally more open to press communication than other stakeholder groups (“*NGOs communicate very well*”).

When asked about a potential conflict of interest or “forbidden topics”, journalists' responses varied from not experiencing censorship (“*I've never had to deal with the situation”; “I never felt that I'm not independent*”), to facing pressure from industry, especially from newspaper sponsors (“*it was the only newspaper in Parma owned by the industrialists of Parma. So, there were some things that I couldn't even propose*”). Investigations into politicians present another notable scenario, potentially endangering journalists (“*companies, organizations, politicians, texting you that there are some problems, some topic, or you should say something this way or that way*”; “*journalist wants to write about a mayor of a city these authorities tell them, do you remember that your daughter is in our school?*”).

#### 3.2.3Impact of the COVID-19 pandemic

Interviewed journalists generally found the pandemic period challenging. Lockdowns, with their work-from-home policies and online meetings, significantly hindered networking opportunities (“*so the opportunity to meet new interviewees was blocked*”).

During the pandemic, COVID-19 dominated newspaper topics (“*the focus shifted overwhelmingly to COVID-19*”; “*readers primarily sought information on COVID-19*”) and more general health-related topics (“*I think there were more articles about health*”; “*the articles related to depression and anxiety were more interesting because the stats show that the cases of anxiety and depression have increased since 2020*”). Various topics were often interwoven with the pandemic (“*even the sports page, for example, spoke of COVID*”). On a smaller note, there was also a focus on the pandemic's impact on environmental issues like plastic pollution (“*tried to show how these single use items can be dangerous for our planet*”). On one hand, environmental concerns were deemed less critical (“I *think that our readers were not interested in this; still when people were dying and sick, this was more important than ecological issues*”), however, interest in environmental issues appeared to grow independently of the pandemic (“*the interest in the environment is growing year after year, I'm not sure that COVID had such an impact on that*”).

According to the majority of the journalists, the pandemic has made connecting and cooperating with stakeholders easier (“*perhaps it has also become a little easier to get in touch with people who perhaps seemed unreachable before the COVID*”), even though at the very beginning it seemed to be harder to get in touch with them (“*initially, contacting stakeholders became more challenging*”).

#### 3.2.4Criticism & areas for improvement

Many respondents believe the press plays a crucial role in society, ranging from the responsibility to report accurate facts (“*I feel the weight of this, because I realize that what I write, and what I then edit is seen by many people*”), to exerting significant influence on public opinion (“*yes, I think it has a really big influence on opinion*”), although some interviewees believe their articles do not significantly impact change (“*I don't consider that my role is to make people change*”).

In the opinion of our respondents, respondents primarily call for innovation in journalism to induce change, such as: (a) increasing media independence (“*so the most important thing is independence in media*”), (b) reducing regulatory restrictions (“*and then regulation, that has reduced the freedom of the press*”), (c) allocating more time for drafting articles (“*if you have to go out in the newspaper tomorrow, I can take up to today's afternoon to get well-informed because then it's a whole production chain*”). Such changes would facilitate thorough fact-checking (“*sometimes journalists want to prepare an article in a very short time and don't want to waste time on some problem and show us only a piece of it*”). Respondents also noted the need for improved salaries to enhance journalists' working conditions (“*to give some money to media to give an opportunity to journalists to have time, have money, to dig deeper*”).

Interviewees attribute the low popularity of environmental topics mainly to insufficient time for thorough investigation (“*they have many journalists who are not in a hurry to work and therefore do not have to write an article in one day alone*”), and the journalists' limited knowledge on the subject (“*journalists themselves know nothing about environmental problems*”). Interviewees proposed several solutions, such as writing more often about it (“*in my opinion, we should frame the environmental problem as COVID*”), including writing more positive environmental stories (“*talking about environmental issues is done only if there are numbers to give or bad news. This is kind of what needs to change*”). Interviewees also suggested the press should engage the public more effectively in the proposed topics (“*I believe that we should use somehow this opportunity which is created by the Internet to make it more interactive and more open, not transferring knowledge from the top down*”), thus preventing the formation of echo chambers (“*people are on the right and on the left and there's nothing in the middle*”), whose foundation is so called cherry-picking of information (“*but they are looking for confirmation of their opinion. That is why they choose particular media*”). Additionally, there should be efforts to motivate readers to take action (“*I have sometimes this feeling that we are talking a lot and doing not enough*”) and try to get closer to the audience by showing the direct impact of environmental threats, positioned equally to other topics, such as the pandemic (“*and write about their cases, their everyday living. To be closer to the audience*”).

## 4 Discussion

Understanding societal perceptions of plastic and its environmental impact is crucial to driving behavioural changes to address plastic pollution. Mainstream media plays a crucial role in informing the public and shaping opinions on emerging environmental issues, influencing public and policy discourses, thereby contributing to awareness and encouraging political action [23]. Nevertheless, the proliferation of fake news and misinformation presents an escalating challenge that could undermine trust in journalism and science [54,55]. Our research confirms that journalists writing about SUP are aware of the dangers of these phenomena and are taking precautions and trying to organize their work such as to allow for in-depth, balanced, and factual environmental journalism.

Our findings reveal that stakeholder engagement is prevalent and valued in journalistic practices. Politicians and industry representatives were the most frequently mentioned stakeholders. The COVID-19 pandemic has led to increased stakeholder engagement, especially related to scientists and recyclers. In Germany, the presence of NGOs increased. This indicates that environmental journalists must adeptly navigate between very different areas of public life, including understanding the realities of economics and politics. Thus, the social dynamics encompass scientists, the public, political actors, and other stakeholders in the circular economy of SUP. Our study corroborates Henderson and Green's [23] observation of a ‘complex interplay’ among stakeholders in SUP discourses, while pointing to the slight predominance of politicians and producers over experts and scientists. This dynamic communication landscape features varied interest groups pursuing distinct objectives. Journalists face the challenge of accurately and precisely describing scientific findings, particularly essential in view of the COVID-19 pandemic [56].

Reporting on plastics significantly declined in the media of the investigated countries following the COVID-19 pandemic. Is the coronavirus pandemic pushing the public attention away from the climate crisis? Empirical research confirms this: pandemic concerns have overshadowed environmental issues in public discourse [57,58]. This is consistent with the findings of [32,37]. Nevertheless, we, like the cited authors, view the decline in interest in SUP not as a lasting change but rather as a temporary shift, presenting descriptive observations.

Our findings indicate that media discourses in the selected countries are more focused on SUPs, whether generally or specific items, rather than on plastics broadly. The Single Use Plastics Directive Implementation Assessment Report [59] indicates that many EU countries have progressed in eliminating several SUP items from their markets, while others are still lagging behind. In our study, the most mentioned SUP items were bottles and packaging, two SUP categories which were not included in the new EU regulations. This discrepancy between public perception, as reflected by the media, and regulatory actions highlights a significant finding (via the media) perceives as the ‘plastics problem’ and what the regulator is doing. We observed an increase in the mention of COVID-19 related SUP items like masks and gloves, whereas mentions of other items such as grocery bags, trash bags, bottles, and straws decreased. Specifically, mentions of bottles and straws decreased in France, while in Germany, there were more mentions of laboratory utensils and fewer of grocery bags and trash bags. These findings align with the assessment report mentioned earlier, as France presents a correct implementation, Germany's and Italy's implementations are underway or incomplete, while Poland shows no implementation at all [59].

In severe global crises, life and health issues become paramount [60]. The pandemic quickly relegated environmental and climate protection to a lower priority amid recession concerns. Societal willingness to engage in climate protection naturally waned in the face of more immediate existential concerns [61]. The pandemic exacerbated the negative impact of pre-existing social inequalities by hitting the socially excluded and disadvantaged groups harder [62]. Furthermore, progress towards environmentally friendly behavioral changes appeared to stall (i.e., usage of multi-use products instead of SUPs), seemed deferred [63]. There remains a gap in public understanding and perspective that the environmental crisis (e.g., plastic waste increase) is significantly interlinked with their secure, well being and health state [64].

Yet, COVID-19 demonstrates how certain events can catalyze significant social and economic changes. Companies instrumental in maintaining operations during the pandemic can similarly contribute significantly to climate change efforts. For the European economy to truly become a circular economy, all stakeholders need to be engaged: public authorities, businesses, trade unions, consumers and civil society as a whole.

Science journalism offers two theoretical entry points. One can either come from scientific background and switch to the media industry, or work in journalism and focus on scientific topics. Journalists frequently lack essential scientific understanding and expertise, especially when it comes to interpreting and classifying scientific studies. In science, on the other hand, the priority, for many reasons, is usually on the research *per se* rather than on journalistic (scientific) communication. A new strategy is needed not only to train journalists but also to make journalism more appealing to researchers. For most scientists and scientific institutions, it is apparently less necessary for survival than for politicians and brand owners to seek the limelight of the public. Consequently, science communication has remained less developed than corporate communications and political campaigning. There is a strong gap between companies and politics on one hand, and the scientific community on the other hand, in the perception of whether, and to what extent, self-promotion must be carried out and public attention is even necessary [65,66].

According to our interviewees, companies, associations, and politicians continue to steer reporting in their favour or to obstruct research. In fact, attempts by PR agencies or PR departments to exert influence have increased, and the line between PR and journalism is becoming increasingly blurred [67,68]. As a result, a large part of journalistic content is based on public relations efforts by companies, ministries, authorities, and other institutions. PR material is increasingly finding its way into the media unfiltered [69]. The interviewees we interviewed, pointed out the practical convenience of working with ready-made materials sent to the newsroom from outside. McKinnon [33] came to similar conclusions, showing significant similarities in the press materials of competing French newspapers.

The interviews conducted with journalists also reveal certain dissimilarities in journalistic practices in the four countries we studied, compared to the findings of Fauzi and Nugrahani [36] on materials from Nordic media. Our interviewees placed great emphasis on the balance of information provided and caution in supporting a political agenda. Nordic media, on the other hand, are more inclined to actively fight for strong pro-environmental policies in Europe.

Furthermore, the more jobs the publishers cut in their editorial offices and the more the work for the remaining employees intensifies, and the greater the gateway for messages from PR and lobbyism in the media, the less it is possible to check predetermined information, question interests and search for topics independently. The COVID-19 crisis has had a significant impact on journalists' work, as the expectations and demands on reporting have increased substantially, necessitating rapid rethinking and action. The crisis has brought about fundamental changes in the editorial practice: the majority of editorial offices have been working from home, and the workflow had to be largely switched to online or handled by phone, which has extended the coordination processes accordingly.

The study has a few limitations. In the media content analysis section, we focused on the quantitative representation of the different SUP categories without examining the context in which these categories appeared. This limitation was due to the linguistic and cultural diversity of our research sample. Therefore, we were not able to compare detailed framing strategies of the SUP issue by the media, although this is a prominent thread in many other studies.

Another limitation of the research is the method of in-depth interviews used to study journalistic practices. It is reasonable to assume that journalists' statements may differ in part from the description of such practices obtained through participant observation. Such observation on a sample of four different European countries was beyond our organizational reach, also as a result of the COVID-19 pandemic during which our research was conducted.

Finally, addressing a critical truth knowledge on plastic requires a broad transdisciplinary science communication (popularization) that interfaces in a smoother way with consumers and policy stakeholders. Here, we met obstacles at recruitment stage. Having a detailed data base on journalists potentially focusing on environmental issues, almost none was interested in sharing their experience with us. This requires linking our developing understanding of science popularization needs with a deeper understanding of the research/journalism system that is not operating as a fragmented but linear or even circular models.

## 5 Conclusions

The pandemic has had a small but statistically significant impact on the reversal of interest in plastic pollution in media discourse. Our research shows that this impact is contextual, both at the level of countries and at the level of discourse elements, such as interest in specific types of plastic goods. During the pandemic period, media interest in certain plastic products decreased, while others increased. This leads us to conclude that the pandemic was not an event whose impact on perceptions of the plastic pollution problem can be clearly assessed causally.

Interviews with journalists reveal the nuanced and contingent nature of their work in raising awareness about plastic pollution. Journalists point to problems and dilemmas that are characteristic of qualitative journalism that aims to debate important social issues, not just in times of pandemic and not just on environmental issues. We conclude that the problems of the discourse on single-use plastics should then be explored in case studies that take into account local and situational conditions.

## Funding

The publication was funded by grant number 2020/39/B/HS4/00264, by the 10.13039/501100004442National Science Centre, Poland.

## Data availability statement

The data used in interviews is confidential.

The data used in media content analysis is presented in the appendices.

## CRediT authorship contribution statement

**Aleksandra Krawczyk:** Writing – original draft, Supervision, Project administration, Methodology, Investigation, Conceptualization. **Alicja Goc:** Writing – original draft, Investigation, Formal analysis, Conceptualization. **Airis Pellegrini:** Investigation. **Natalia Jaguszewska:** Investigation. **Brenda Olivos Salas:** Writing – review & editing. **Michał Bukowski:** Writing – review & editing. **Małgorzata Grodzińska-Jurczak:** Supervision, Methodology, Funding acquisition, Conceptualization.

## Declaration of competing interest

The authors declare the following financial interests/personal relationships which may be considered as potential competing interests: Malgorzata Grodzinska-Jurczak reports financial support was provided by National Science Centre Poland. If there are other authors, they declare that they have no known competing financial interests or personal relationships that could have appeared to influence the work reported in this paper.

## Figures and Tables

**Table:** 

	Bottles	Packaging	Masks	Cups	Bags (grocery)	Food containers	Straws	Cutlery	Bags (trash)	Gloves	Bags (fruits/vegies)	Laboratory utensils	Plates	Menstrual utensils	ALL
2019	69	28	0	13	10	6	10	4	5	0	4	0	2	0	151
Jun	8	4	0	2	3	1	3	0	1	0	4	0	0	0	26
Jul	13	3	0	4	2	1	5	2	1	0	0	0	0	0	31
Aug	5	3	0	0	2	0	1	0	2	0	0	0	1	0	14
Sep	15	5	0	2	2	0	0	1	0	0	0	0	0	0	25
Oct	7	5	0	1	0	0	0	0	1	0	0	0	0	0	14
Nov	9	4	0	1	1	1	1	0	0	0	0	0	0	0	17
Dec	12	4	0	3	0	3	0	1	0	0	0	0	1	0	24
**2020**	**32**	**29**	**17**	**7**	**6**	**5**	**3**	**3**	**1**	**6**	**0**	**0**	**0**	**0**	**109**
Jan	9	6	0	2	2	0	1	0	1	0	0	0	0	0	21
Feb	3	1	0	2	0	0	0	0	0	0	0	0	0	0	6
Mar	4	1	0	0	0	0	0	2	0	0	0	0	0	0	7
Apr	0	1	2	0	0	1	0	0	0	2	0	0	0	0	6
May	0	2	6	2	0	1	0	1	0	3	0	0	0	0	15
Jun	1	7	5	0	0	0	1	0	0	1	0	0	0	0	15
Jul	2	4	1	0	0	0	0	0	0	0	0	0	0	0	7
Aug	0	1	1	0	1	0	0	0	0	0	0	0	0	0	3
Sep	2	3	2	0	3	1	1	0	0	0	0	0	0	0	12
Oct	1	0	0	0	0	0	0	0	0	0	0	0	0	0	1
Nov	2	1	0	1	0	1	0	0	0	0	0	0	0	0	5
Dec	8	2	0	0	0	1	0	0	0	0	0	0	0	0	11
**2021**	**14**	**7**	**6**	**3**	**3**	**8**	**0**	**3**	**1**	**1**	**1**	**4**	**2**	**1**	**54**
Jan	3	2	2	1	1	3	0	0	0	0	1	1	0	0	14
Feb	2	1	0	0	0	1	0	0	0	0	0	0	0	0	4
Mar	4	1	1	1	0	0	0	0	0	0	0	0	0	0	7
Apr	1	1	0	0	1	1	0	1	0	0	0	1	0	1	7
May	1	2	3	1	1	2	0	2	1	1	0	2	2	0	18
Jun	3	0	0	0	0	1	0	0	0	0	0	0	0	0	4
**Sum**	**115**	**64**	**23**	**23**	**19**	**19**	**13**	**10**	**7**	**7**	**5**	**4**	**4**	**1**	**314**

**Table:** 

	Politicians	Industry	NGOs	Scientists	Producers	Recyclers	Waste management	Consumers	Designers	ALL
2019	119	101	61	40	17	12	12	11	4	377
Jun	19	15	14	8	4	5	4	0	0	69
Jul	32	15	13	9	2	1	5	2	0	79
Aug	14	12	8	4	3	1	0	1	3	46
Sep	14	9	8	7	0	1	0	3	0	42
Oct	8	20	4	7	4	3	1	2	1	50
Nov	13	17	4	1	2	1	0	0	0	38
Dec	19	13	10	4	2	0	2	3	0	53
**2020**	**89**	**88**	**53**	**49**	**32**	**17**	**14**	**13**	**3**	**358**
Jan	13	11	8	6	4	0	0	2	0	44
Feb	9	8	4	5	5	0	1	0	0	32
Mar	6	7	1	4	3	0	0	1	1	23
Apr	6	1	4	4	1	1	2	0	0	19
May	10	5	6	4	2	2	4	2	0	35
Jun	10	8	13	5	4	2	3	4	1	50
Jul	4	10	4	4	2	3	1	1	0	29
Aug	5	6	2	3	2	1	1	0	0	20
Sep	7	11	5	1	4	3	1	0	0	32
Oct	4	5	1	4	0	1	0	1	0	16
Nov	4	8	1	4	3	2	0	0	0	22
Dec	11	8	4	5	2	2	1	2	1	36
**2021**	**40**	**45**	**16**	**21**	**11**	**10**	**5**	**1**	**0**	**149**
Jan	13	6	5	4	2	1	1	0	0	32
Feb	7	6	0	2	1	1	1	0	0	18
Mar	2	9	2	3	1	1	2	0	0	20
Apr	5	4	2	5	3	2	0	0	0	21
May	10	17	7	6	4	4	1	1	0	50
Jun	3	3	0	1	0	1	0	0	0	8
**Sum**	**248**	**234**	**130**	**110**	**60**	**39**	**31**	**25**	**7**	**884**

**C.1:**
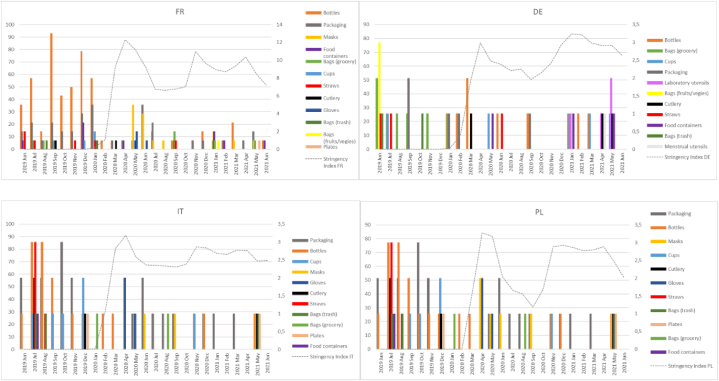
SUP item specifications per country (France, Germany, Italy, Poland).

**C.2:**
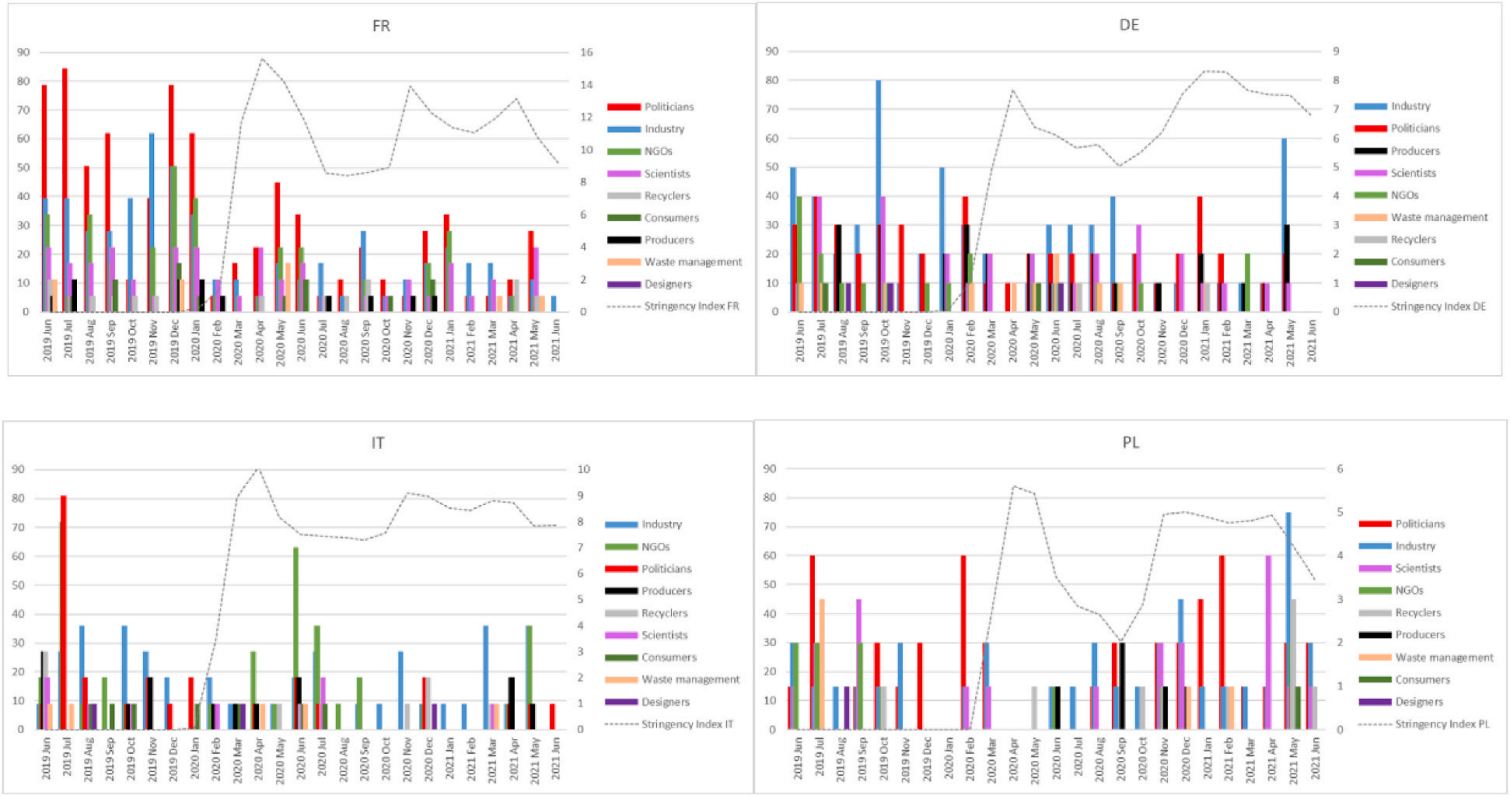
Stakeholder engagement per country (France, Germany, Italy, Poland).

**D.1:**
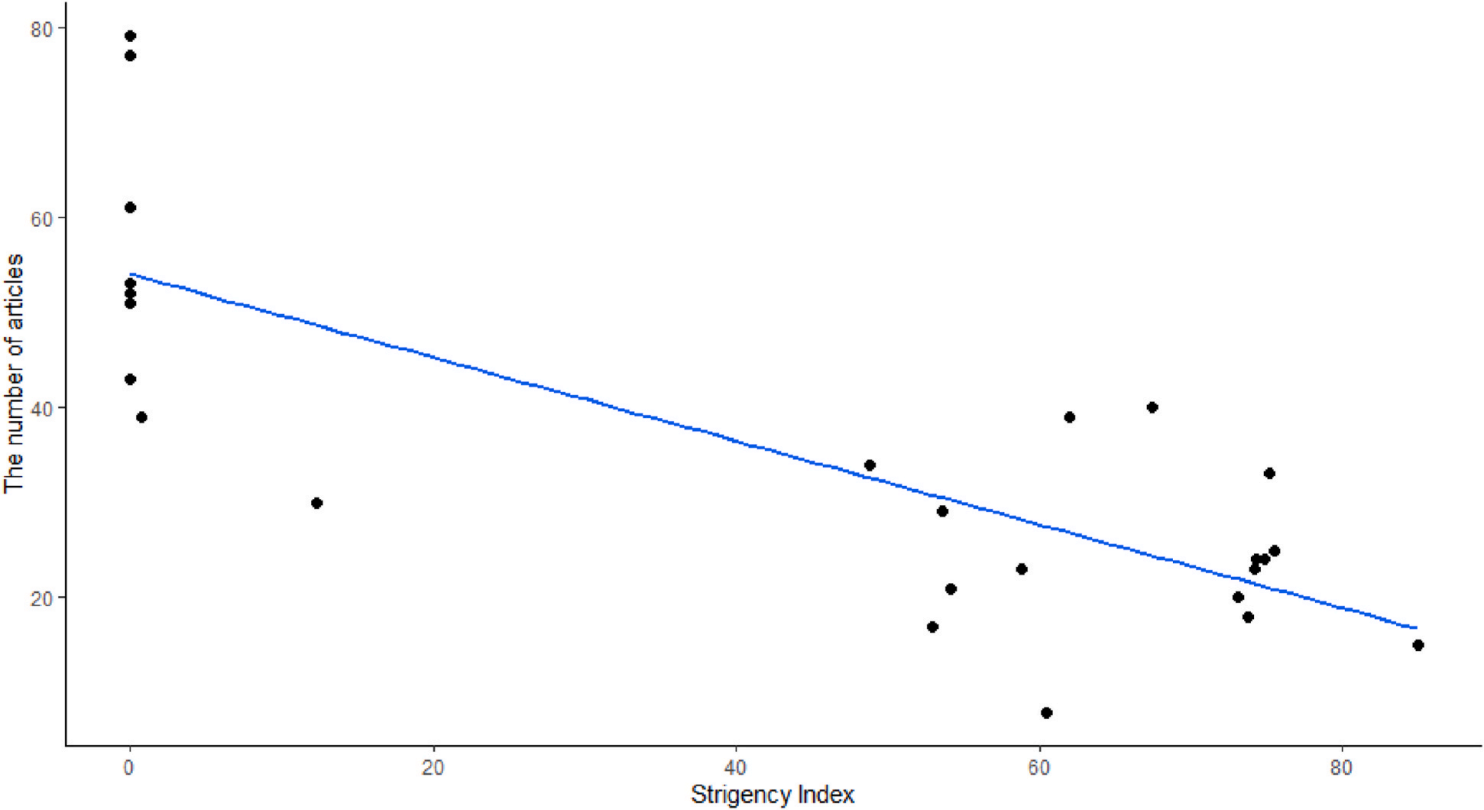
Correlation between the stringency index and the number of articles (∼p = 0.0095, ∼ρ = −0.26).

**D.2:**
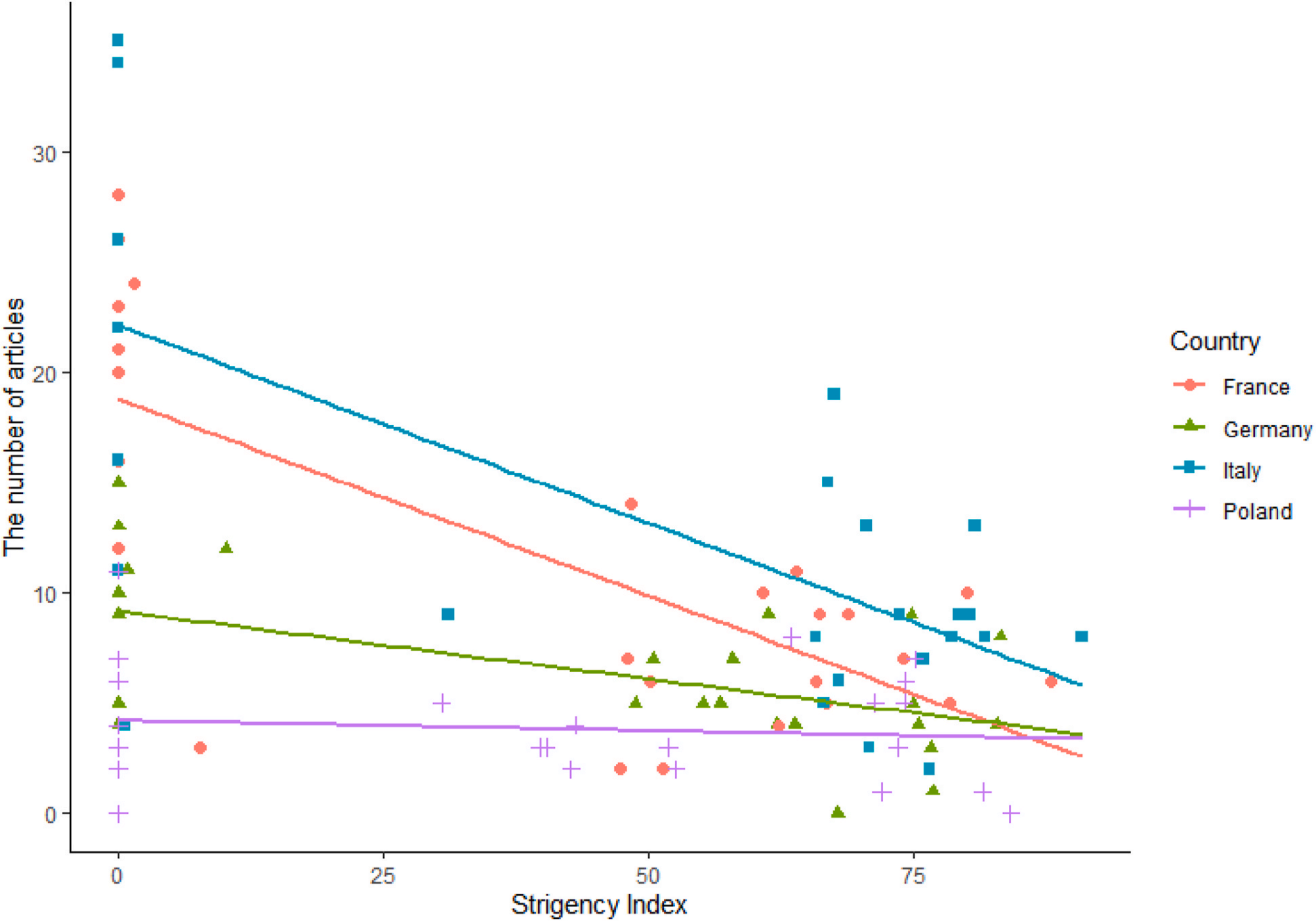
Correlation between the stringency index and the number of articles in different countries.

**D.3:**
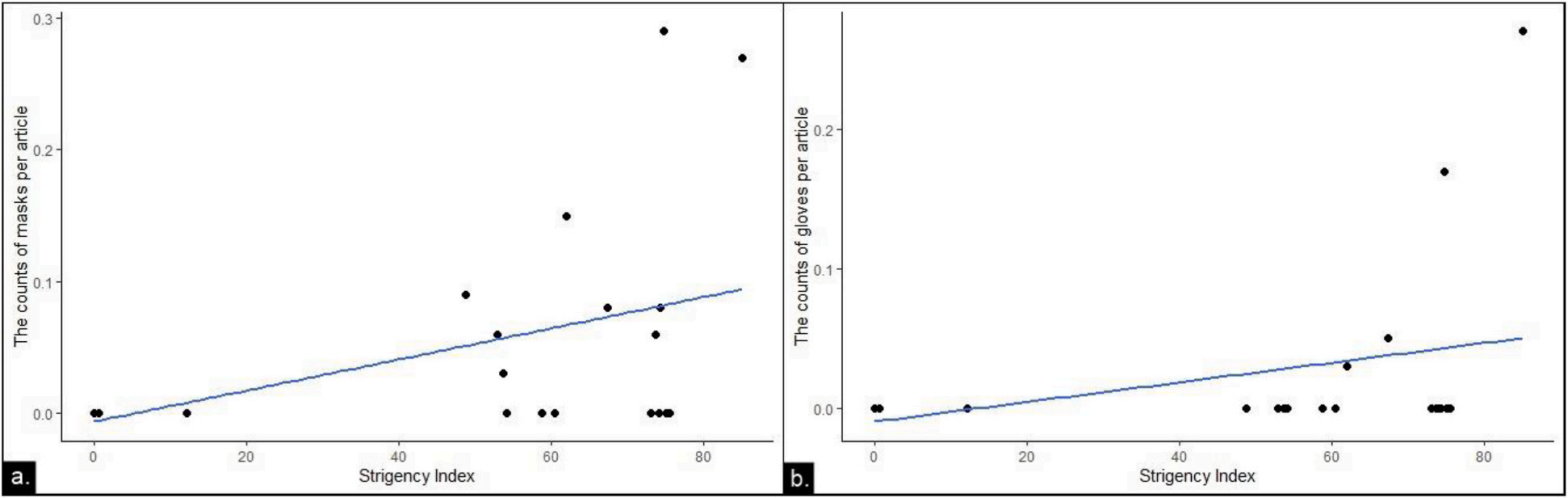
Positive correlation between the stringency index and the monthly counts of (a.) masks per article, (b.) gloves per article

**D.4:**
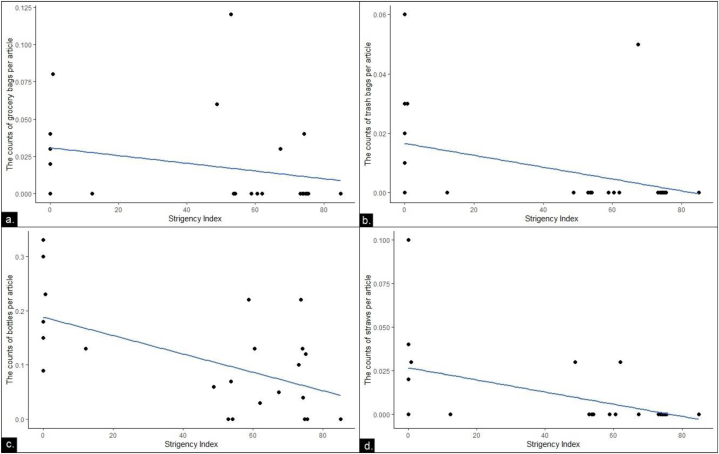
Negative correlation between the stringency index and the monthly counts of (a.) grocery bags per article, (b.) trash bags per article, (c.) bottles per article, (d.) straws per article

**D.5:**
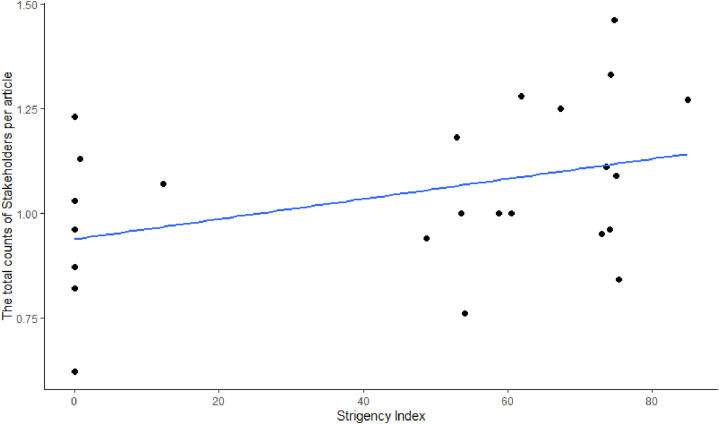
The total counts of stakeholders mentioned monthly per article over the stringency index

**Figure:**
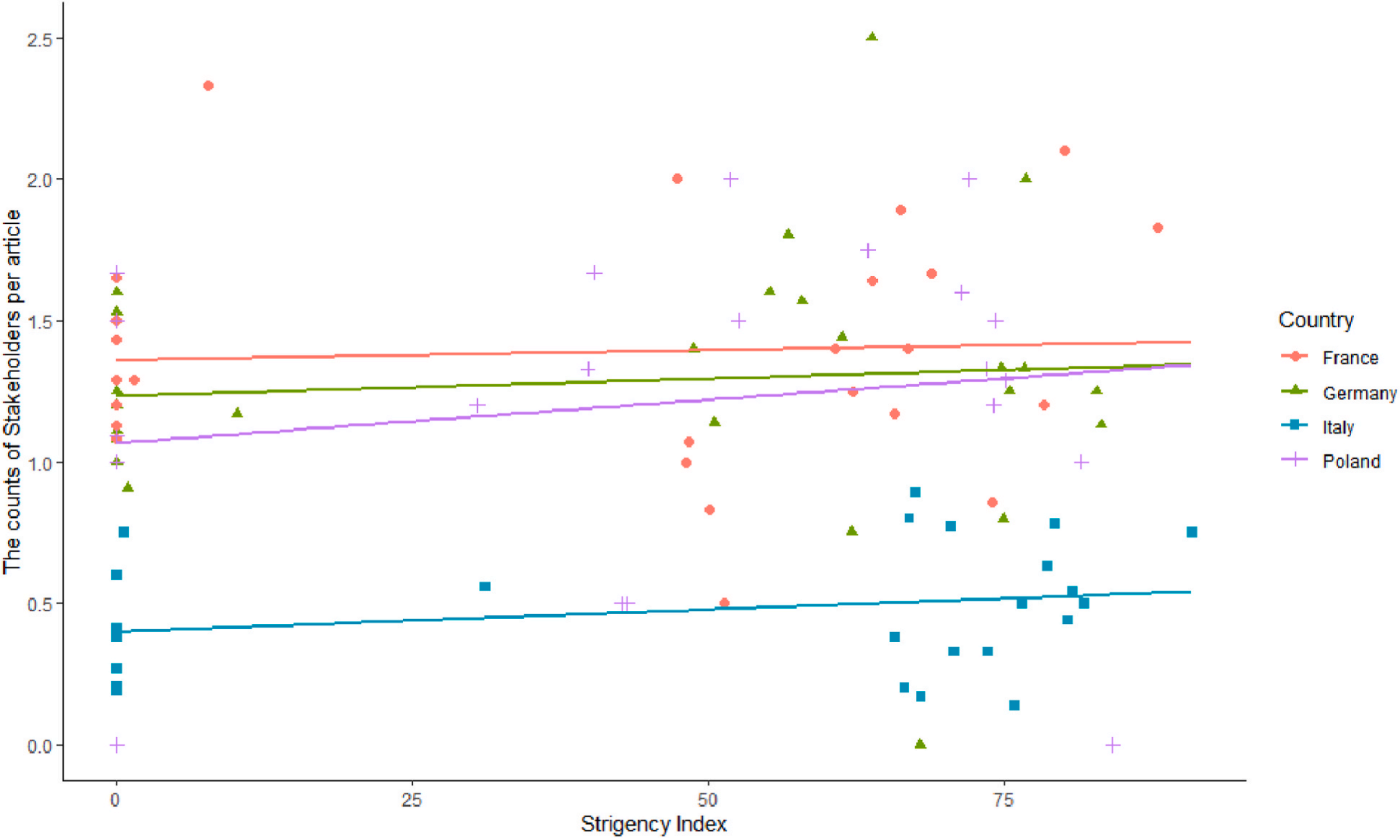
D.6Counts of stakeholders mentioned monthly per article per country over the stringency index

**D.7:**
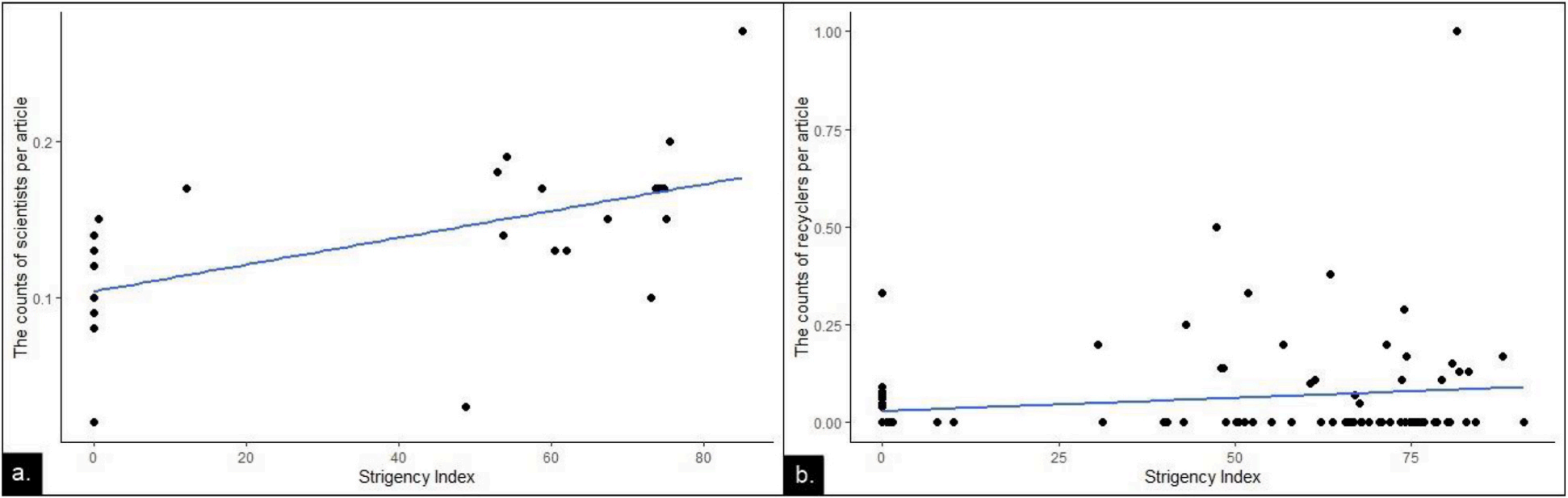
Positive correlation between the stringency index and the monthly counts of (a.) scientists mentioned per article, (b.) recyclers mentioned per article
